# The Behavior of Entanglements in a Polymer Gel under Isotropic Swelling

**DOI:** 10.1021/acs.macromol.5c03142

**Published:** 2026-04-24

**Authors:** Zefan Shao, Rui Ji, Qihan Liu

**Affiliations:** Department of Mechanical Engineering and Materials Science, 6614 University of Pittsburgh, Pittsburgh, Pennsylvania 15213, United States

## Abstract

Cross-links and entanglements are two molecular features in a polymer network that transfer the macroscopic load to individual polymer chains. Existing theories assume that (1) entanglements can slide and lead to distinct elastic responses compared to cross-links in both isotropic and anisotropic deformation, and (2) permanent entanglements form during network synthesis and transient entanglements form when deswelling the polymer network from the synthesis state. Using the constrained swelling test to directly measure the osmotic pressure, we show that (1) entanglements and cross-links behave identically under isotropic swelling, indicating no entanglement sliding, and (2) the osmotic pressure follows the power law of transient entanglement in both swollen and deswollen states, indicating transient entanglements also form during network synthesis.

## Graphical abstract

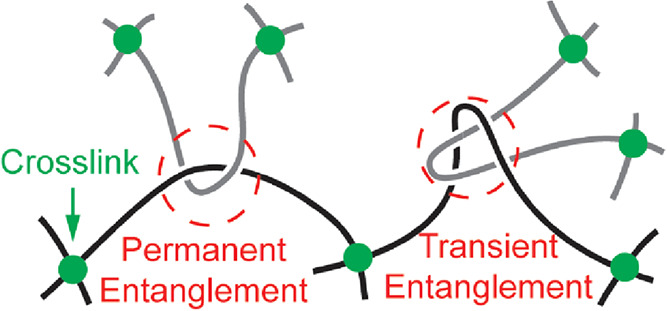

## 1 Introduction

Polymeric gels can swell or deswell in response to stimuli such as temperature, light, electromagnetic field, specific solvents, and various biomolecules, thus often considered smart materials.^1–3^ The spontaneous swelling is invaluable for actuation in hard-to-access sites; important applications include swellable packers in oil wells,^4^ drug delivery systems inside human bodies,^5^ automatic valves in microfluidic chips,^6^ and continuous wound exudate absorption.^7^ The swelling is driven by the osmotic pressure *Π*, which quantifies the suction applied by a gel to a solvent in contact.^8,9^ The higher the *Π*, the stronger the gel absorbs the solvent. The osmotic pressure of a polymeric gel is generally divided into two parts: a mixing part due to the polymer–solvent interaction, *Π*_mix_, and an elastic part due to the polymer chain stretching, *Π*_ela_.^10–15^ Here, the microscopic polymer chain stretching is coupled to the macroscopic deformation through cross-links and entanglements (Figure 1): cross-links permanently connect polymer chains at one site; entanglements prevent polymer chains from crossing each other but allow sliding. Some entanglements are permanently trapped between cross-links, thus can only slide in a limited range and cannot slide out of a chain end or a chain loop to disappear. Other entanglements are not trapped between cross-links, thus only transiently prevent the lateral motion of a polymer chain: once the chain slides away, the entanglement disappears. Note that dangling chains do not carry elastic stress, thus the transient entanglements of dangling chains are excluded in the discussion. Due to the sliding, the elastic response due to entanglements softens with deformation compared to cross-links.^16^ According to existing constitutive models, entanglements should slide under both isotropic and anisotropic deformation. However, this prediction is never verified due to the difficulty of measuring the mechanical response in isotropic deformation: the osmotic pressure *Π*. Here, we use the constrained swelling test to measure *Π* under isotropic swelling and to distinguish between the contributions from cross-links and entanglements through different synthesis conditions. Contrary to existing theories, our measurements show that cross-links and entanglements behave identically in measured *Π*_ela_, suggesting that entanglements do not slide under isotropic deformation. Moreover, existing theory claims that all entanglements are trapped during synthesis. Transient entanglements only form when deswelling a polymer network.^17,18^ Our measured *Π*_ela_ agrees with the scaling of transient entanglements in both swollen and deswelling states, suggesting that transient entanglements form with permanent entanglements during synthesis and persist into swollen states.

**Figure 1:**
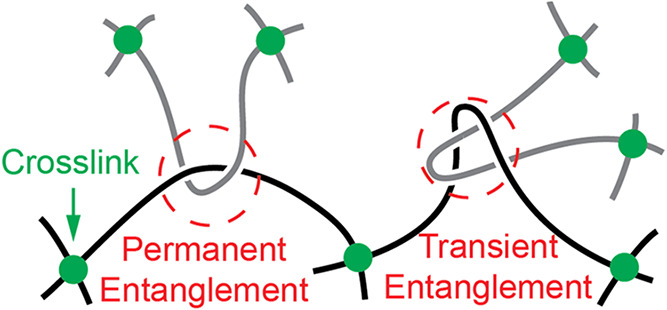
Cross-links are permanent links between two polymer chains. Entanglements are slidable topological constraints that prevent polymer chains from crossing each other. Permanent entanglements are trapped between cross-links. Transient entanglements can pull out from the constrained loop.

## 2 Existing Models of Entangled Polymer Networks

Constitutive models of polymeric gels are commonly formulated through the free energy *W* per reference volume^19^

1 $$W=W_{\mathrm{mix}}\left(\phi \right)+W_{\mathrm{ela}}\left(F\right)$$

Here, *W*_mix_ accounts for the free energy to mix uncross-linked polymer with the solvent, which is solely determined by the polymer volume fraction *ϕ*. *W*_ela_ accounts for the elasticity of the polymer network and is solely determined by the deformation gradient tensor **F**.^20,21^ Additional terms may be added for other physics involved.^10–15^ Since the bulk moduli of polymers and solvents (∼GPa) are orders of magnitude higher than the shear moduli of swollen polymer networks (∼10 MPa or lower), swellable polymer networks can be modeled as incompressible in the absence of solvent exchange.^22^ Then the deformation and swelling are connected through det­(**F**) = 1/*ϕ*. Here, det­(**F**) is also known as the swelling ratio.

In existing constitutive models, *W*_mix_ is commonly assumed to follow the Flory–Huggins model

2 $$W_{mix}\left(\phi \right)=\frac{\mathrm{kT}}{\Omega }\left[\left(\frac{1}{\phi }-1\right)\ln\left(1-\phi \right)-\chi \phi \right]$$

Here, *k* is the Boltzmann constant, *T* is the thermodynamic temperature, Ω is the average volume per solvent molecule, and *χ* is a dimensionless parameter characterizing the mixing enthalpy. When entanglement is considered, *W*_ela_ is commonly divided into two additive parts

3 $$W_{\mathrm{ela}}\left(F\right)=W_{C}\left(F\right)+W_{E}\left(F\right)$$

Here, *W*_C_ accounts for the contribution from cross-links, and *W*_E_ accounts for the contribution from entanglements. For *W*_C_(**F**), while existing models vary on the treatment near the chain extensibility limit, they recover to the neo-Hookean model at moderate deformation^23^

4 $$W_{C}\left(F\right)=\frac{G_{C0}}{2}\sum_{i=1}^{3}\lambda_{i}^{2}$$

Here, *G*_C0_ is the shear modulus in the reference state, which is proportional to the concentration of cross-links. *λ*_1_, *λ*_2_, and *λ*_3_ are the eigenvalues of the deformation gradient **F**. For swellable polymer networks, one additional logarithmic term is suggested by Flory to account for the change in the translational entropy during cross-linking^24^

5 $$W_{C}\left(F\right)=\frac{G_{C0}}{2}\left(\sum_{i=1}^{3}\lambda_{i}^{2}-\ln\left(\lambda_{1}\lambda_{2}\lambda_{3}\right)\right)$$

Numerous models have been proposed for *W*_E_.^16,25^ When modeling the elastic properties of entangled polymer networks, tube models are widely used because of their great success in the rheological studies of entangled polymer melts^26^ and their relatively simple expression of *W*_E_. In a tube model, the constraints from the entanglements on one chain are represented as tubes (Figure 2A). The tube constrains the lateral motion of that chain but allows free sliding along the tube. As the polymer network deforms, the chain is stretched inside the tube, and the tube diameter changes according to the change in the number of entanglements. Here, a decrease in tube diameter corresponds to an increase in the number of entanglements. A network-level tube model is derived by averaging the tube deformation in different orientations. Most tube models average over three principal stretch directions and can be arranged into the following form^27^

6 $$W_{E}\left(F\right)=\frac{2G_{E0}}{\beta^{2}+\gamma^{2}}\sum_{i=1}^{3}\left(\frac{1}{\lambda_{i}^{\beta }}+\lambda_{i}^{\gamma }\right)$$

**Figure 2:**
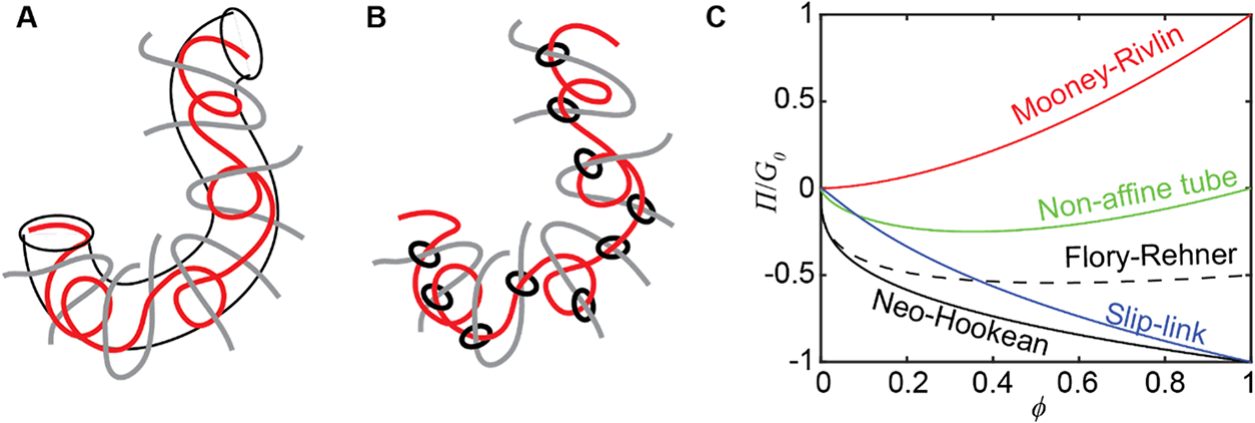
(A) Tube models treat entanglements as a tube constraining the lateral motion of a polymer chain. (B) The slip-link models treat entanglements as slidable links between entangled chains. (C) Normalized osmotic pressure from existing models.

Here, *G*_E0_ is the shear modulus in the reference state due to entanglements, which is proportional to the concentration of entanglements. The parameter *β* dictates how the tube diameter decreases with stretching. If the tube maintains a constant volume when axially stretched, *β* = 1.^28^ If the tube diameter changes affinely with lateral deformation, then *β* = 2. Other *β* values representing different tube behaviors have been discussed, but are rarely used in modeling.^25^ *γ* dictates how polymer chains are stretched along the tube. *γ* = 0 means no stretching in addition to the neo-Hookean model (eq 4). The case of *β* = 2, *γ* = 0, or *γ* = 2 corresponds to the Mooney-Rivlin model, a popular phenomenological model to study entangled polymer networks.^29,30^ Here, the *λ*_i_^*γ*^ terms with *γ* = 2 will lump into *W*_C_ during data fitting. The case of *β* = 1, *γ* = 0 was proposed by Gaylord and Edwards in the early development of the tube models,^28,31^ and is still used in recent models of entangled polymer gels.^27,32,33^ The case of *β* = *γ* = 1, derived by Rubinstein et al., is known as the nonaffine tube model.^34^ Other *γ* values have been used to account for the molecular-level strain amplification caused by network imperfections and chain sliding.^27^

Besides averaging over three principal orientations, there are variations of the tube model using analytically derived 4-chain and 8-chain averaging and numerically integrated full-network averaging over up to 983,040 orientations.^35–39^ Also, the slip-tube model modifies eq 5 with an additional renormalization parameter to account for the chain sliding in the tube.^16^ These variations do not change the expression of eq 5 in the isotropic deformation studied in this paper. Since tube models assume that the tube in each orientation responds independently, they predict the same tube contraction in isotropic and anisotropic deformation.

Slip-link models are also popular in the study of entangled polymer dynamics.^40^ Slip-link models treat each entanglement as a link that can slide along the neighboring chains (Figure 2B). Since each link is individually tracked, slip-link models are often implemented in coarse-grained molecular simulations rather than a continuum constitutive model. A free-energy density in terms of macroscopic deformation is rarely formulated. A free energy density dating back to the 1980s^41,42^ is the only one that is used in modeling entangled polymer networks today^43–45^

7 $$W_{E}\left(F\right)=\frac{G_{E0}{\left(1+\eta \right)}^{2}}{2}\sum_{i=1}^{3}\left(\frac{\left(1+\eta \right)\lambda_{i}^{2}}{1+\eta \lambda_{i}^{2}}+\ln\left(1+\eta \lambda_{i}^{2}\right)\right)$$

Here, *η* describes the amount of entanglement sliding. *η* = 0.2343 is suggested by assuming that each slip-link can slide half the distance between two cross-links/entanglements.^42^ *η* is deformation-independent, indicating that the sliding behavior is identical in isotropic and anisotropic deformation.

The osmotic pressure can be derived from the free energy through^30^

$$\Pi =-\frac{\partial W}{\partial \left(1/\phi \right)}$$ 8

Then, according to eqs 1 and 3, we can similarly split the osmotic pressure using *Π* = *Π*_mix_ + *Π*_ela_ and *Π*_ela_ = *Π*_C_ + *Π*_E_. By eqs 4–7, we know *Π*_C_ ∝ *G*_C0_ and *Π*_E_ ∝ *G*_E0_. We plot the dimensionless *Π*_C_/*G*_C0_ and *Π*_E_/*G*_E0_ in Figure 2C. For the osmotic pressure due to cross-links *Π*_C_, the neo-Hookean model (solid black curve, eq 4) always gives a negative *Π*_C_, i.e., driving the deswelling. The neo-Hookean *Π*_C_ monotonically decreases with polymer volume fraction *ϕ*; i.e., the deswelling effect strengthens with deswelling. After the term for translational entropy in the Flory-Rehner model is added (dashed black curve, eq 5), *Π*_C_ is less negative because the translational entropy prefers swelling. The effect of this additional term is only significant at high *ϕ*, i.e., less swollen states.

For osmotic pressure due to entanglements *Π*_E_, if the stretch along the tube is neglected in the tube model (eq 6 with *γ* = 0), the lateral constraint of the tube gives *Π*_E_ > 0, driving the swelling. Here, the red curve corresponds to the Mooney-Rivlin model with *β* = 2, *γ* = 0. If chain stretching along the tube is sufficiently strong, *γ* ≥ *β*, then *Π*_E_ is always negative, driving the deswelling. Here, the green curve corresponds to the nonaffine tube model with *β* = *γ* = 1. Compared to the tube models, the slip-link model (blue curve, eq 7) leads to a value of *Π*_E_/*G*_E0_ closer to the neo-Hookean *Π*_C_/*G*_C0_. The slippage does lead to a less negative osmotic pressure compared to the neo-Hookean case, implying a weaker driving force to deswell the gel.

## 3 Constrained Swelling Tests to Characterize the Osmotic Pressure *Π*

The model predictions in Figure 2C have never been experimentally verified due to the difficulty of characterizing the osmotic pressure in a polymer network. Conventional mechanical tests of polymeric gels involve measuring the reaction force of a gel sample against certain constraints, such as the stretch between two grips,^46^ the compression between two parallel plates,^47^ and the indentation under an indenter.^48^ These constraints necessarily induce anisotropic deformation. Then, the osmotic pressure must be derived from the measurements using an elastic constitutive model to remove the deviatoric contribution.^20^ In the absence of accurate constitutive models, this approach cannot derive accurate osmotic pressure. To avoid this difficulty, we developed the fully constrained swelling test (Figure 3).^9^ Here, a thin piece of stress-free polymeric gel is tightly fitted between a rigid die and a dense, rigid mesh in its stress-free state. The setup fully constrains the deformation of the gel but allows solvent exchange through the mesh. Since the gel experiences no deformation relative to its stress-free state, the push against the constraint solely comes from the osmotic pressure *Π*, which is directly measured by the load cell.

**Figure 3:**
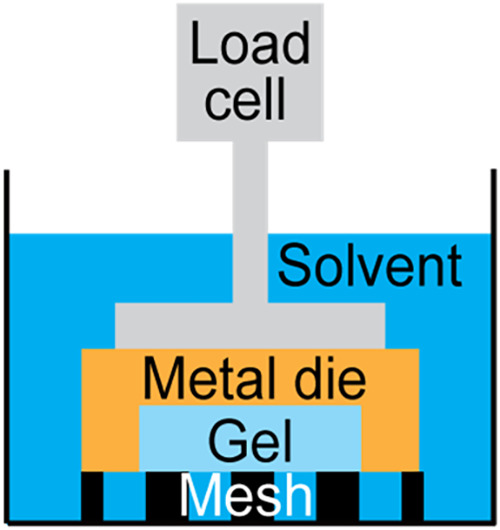
Schematic of the constrained swelling test.

Here, we use a stainless-steel die of diameter *D* = 20 mm and thickness *t* = 1 mm for the constrained swelling test. We synthesized polyacrylamide polymer networks through free radical polymerization using *N*,*N*′-methylenebisacrylamide as the cross-linker and ammonium persulfate as the initiator. Monomer, cross-linker, and initiator are mixed with different initial polymer volume fraction *ϕ*_0_ and cross-linker-monomer ratio *C*. Based on the targeted *ϕ*, a plastic die of diameter (*ϕ*_0_/*ϕ*)^1/3^ *D* and thickness (*ϕ*_0_/*ϕ*)^1/3^ *t* is made. The precursor solution is cured in the plastic die and either dried in the air or swelled in water to reach roughly 1.1 *ϕ* so that the gel is slightly smaller than the stainless-steel die. The gel is then fitted into a stainless-steel die and submerged underwater. A preload of roughly 90% of the final osmotic pressure is applied to compress the stainless-steel die against the metal mesh. This precompression closes any potential gap among the load cell, die, mesh, water container, and the substrate of the compression tester. The setup is then held in place with the load cell monitoring the stress (Figure 3). The hydrogel gradually absorbs water and fully fills the space within the die. Subsequent swelling pushes against the die, leading to an increase in the measured pressure above the precompression. When the water content homogenizes within the hydrogel layer, the measured pressure reaches a plateau, corresponding to the osmotic pressure of the hydrogel. Afterward, the hydrogel samples were taken out and weighed. If the final weight of the gel is more than 5% higher than the expected weight based on the die dimension, which indicates insufficient constraining, then the test is discarded. If the weight change is less than 5%, then the hydrogel is fully dried in a desiccator for over a week. Since some unreacted monomers diffuse out from the hydrogel during the swelling test, the final weight is slightly lower than the original monomer + cross-linker weight in the precursor. The weight loss depends on the polymer volume fraction during synthesis and is roughly 7.5% for *ϕ*_0_ = 0.07 and 4.5% for *ϕ*_0_ = 0.41. The polymer volume fraction *ϕ* of the measurement is then adjusted by the weight loss to reflect the slightly lower *ϕ* value than the target value. The samples are then stored in a large tank of water for 1 week and dehydrated again. Negligible weight change was observed, suggesting that most uncross-linked monomers/oligomers are removed by the end of the constrained swelling test, and our measurements should not be affected by these defects.

## 4 Separating Different Contributions in Osmotic Pressure

We have measured *Π*_mix_ of polyacrylamide hydrogels by synthesizing softer and softer gels to isolate the elasticity-independent part of Π in a previous study.^9^ The data is replotted in Figure 4A,B. The data can be fitted by the Flory–Huggins model with *χ* = 0.46 (eq 2), assuming room-temperature *kT* = 4.1 × 10^–21^ J and water molecular volume Ω = 3.0 × 10^–29^ m^3^. This result is noteworthy in a few ways: (1) Our measurements can be fitted by the Flory–Huggins model over a wide concentration range. In contrast, existing measurements in hydrogels often conclude that a concentration-dependent *χ* is needed.^47^ ,^49^ ,^50^ This is because these studies assumed the *W*_ela_ of the Flory-Rehner model (eq 5). Since this *W*_ela_ is not accurate, its error is reflected in *χ*. (2) Our *χ* = 0.46 is lower than the *χ* = 0.49 measured from polymer solutions of very low concentration (*ϕ* < 1%).^51,52^ Given that the Flory–Huggins model is only expected to work for concentrated polymer solutions,^30^ the measurements at these low concentrations should be interpreted differently. (3) Both the de Gennes scaling theory, *Π*_mix_ ∝ *ϕ* ^2.3^ and the Flory–Huggins model (eq 2) have been used for modeling the swelling of polymer gels at similar polymer volume fractions.^10,47,49,50,53–56^ While the *Π*_mix_ ∝ *ϕ* ^2.3^ scaling is expected to be valid for semidilute solutions and the Flory–Huggins model is expected to be valid for concentrated solutions,^30^ the transition point between these two regimes is rarely determined. Here, we find that although the scaling *Π*_mix_ ∝ *ϕ* ^2.3^ may perform acceptably on a log–log plot (Figure 4A), Flory–Huggins theory performs better in terms of the absolute error (Figure 4B). Since the constrained swelling test on cross-linked gels characterizes much higher osmotic pressure (∼100 kPa to ∼10 MPa) than the conventional membrane osmometry of polymer solutions (∼10 Pa to ∼100 kPa),^57^ this observation does not conflict with existing studies that validate de Gennes scaling theory at much lower polymer volume fraction *ϕ*.

**Figure 4:**
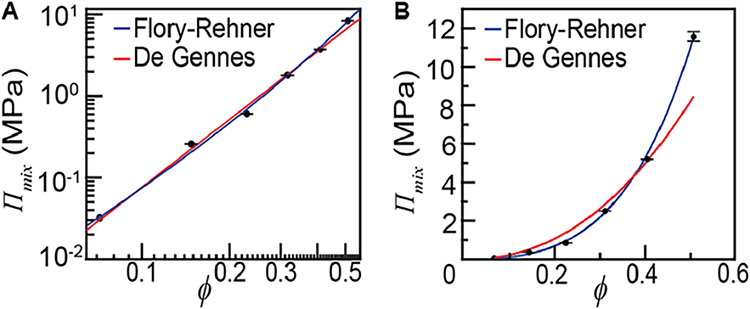
(A) Log–log plot of *Π*_mix_(*ϕ*) shows decent agreement with both the Flory-Rehner and the de-Gennes model. (B) Linear-scale plot of *Π*_mix_(*ϕ*) shows that the Flory-Rehner model outperforms the de-Gennes model. Error bars represent the standard error of the mean for samples of *n* = 9.

With this *Π*_mix_, we can determine *Π*_ela_ by *Π* – *Π*_mix_. We synthesized polyacrylamide hydrogels following the same procedure to study *Π*_ela_. To differentiate the contributions from cross-links and entanglements, we synthesize gels with different densities of cross-links and entanglements and compare their osmotic pressure at identical values of *ϕ*. We control the cross-link density in the polymer network by tuning the ratio between the cross-linkers and monomers in the precursor solution, *C*. We controlled the entanglement density by synthesizing the gel at different initial polymer volume fractions *ϕ*_0_. Existing studies show that a higher *ϕ*_0_ leads to a higher entanglement density.^58–60^ Since free radical polymerization does not proceed perfectly, *C* generally does not equal the ratio between cross-links and monomers in the formed polymer network,^61^ and *ϕ*_0_ also affects the cross-link density.^62^ Nevertheless, a higher *C* leads to a higher cross-link density, and *ϕ*_0_ affects entanglements more than the cross-link density.

We compared the combinations of three cross-linker ratios, *C* = 0.01%, 0.5%, 2%, and three initial polymer volume fractions, *ϕ*_0_ = 0.07, 0.23, and 0.41. In all cases, a higher final polymer volume fraction *ϕ* leads to a higher osmotic pressure *Π*, reflecting that the drier gel is more eager to absorb solvent (Figure 5A). When either *C* or *ϕ*_0_ is fixed, increasing the other lowers the osmotic pressure *Π*, indicating that both cross-links and entanglements drive deswelling. Consequently, tube models without the stretching effect (*γ* = 0) cannot be valid. Moreover, we find that increasing *ϕ*_0_ and increasing *C* can have identical effects, as summarized in Table 1. The overlapping suggests that cross-links and entanglements behave identically under isotropic deformation, disagreeing with existing models reviewed earlier. Yet if we look at the pure shear stress-stretch data of the same group of samples (Figure 5B), we do notice the typical difference between entanglements and cross-links: (1) entanglements lead to softening at large stretch compared to cross-links, which produces a linear *s*-*λ* relation based on eq 4, and (2) entanglements lead to higher failure stretch. This shows that the difference between cross-links and entanglements only shows up in anisotropic deformation. In other words, entanglements do not slide under isotropic deformation. This is reasonable because isotropic deformation increases the tension in all polymer chains proportionally. There is no bias to drive chain sliding.

**Figure 5:**
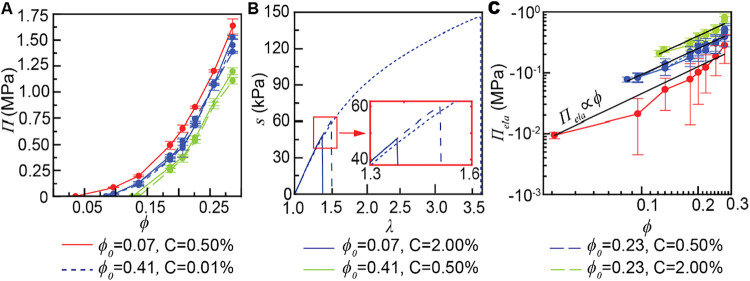
(A) Osmotic pressure (*Π*) of networks synthesized under different conditions shows that more cross-links (higher *C*) or entanglements (higher *ϕ*_0_) reduce the osmotic pressure *Π*. Different ratios of entanglements and cross-links lead to identical *Π*. The *Π* = 0 points are obtained by free swelling. Other points are obtained by fully constrained swelling. Error bars represent the standard error of the mean for samples of *n* = 3. (B) Different ratios of entanglements and cross-links lead to distinct relations between nominal stress *s* and stretch *λ*. (C) Elastic contribution to the osmotic pressure, *Π*_ela_ = *Π* – *Π*_mix_, shows a nearly linear dependence on *ϕ* (solid black lines), following the scaling of transient entanglements. For all figures, error bars represent the standard error of the mean, combining the error from *Π* in (A) and *Π*_mix_ in Figure 4. Colored solid lines are to guide the eye.

**Table 1:**
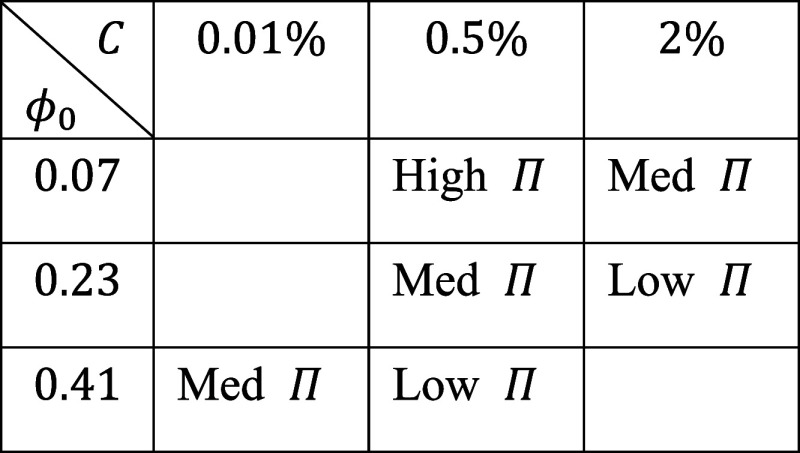
Different Combinations of *C*, *ϕ*_0_ Resulting in Indistinguishable *Π*

Using *Π*_ela_ = *Π* – *Π*_mix_, we can isolate the elastic response from the polymer network (Figure 5C). Since *Π* is dominated by *Π*_mix_ at high *ϕ*, the error of *Π*_ela_ is larger at higher *ϕ*. While cross-links and entanglements behave identically in Figure 5C, the corresponding *Π*_ela_ does not follow the classical neo-Hookean model (eq 4) or the Flory-Rehner model (eq 5). The measured *Π*_ela_ decreases almost linearly with *ϕ* while both the neo-Hookean model and the Flory-Rehner model predict a slower decrease in *Π*_ela_ at higher *ϕ* (Figure 2C). In contrast to *Π*_ela_ ∝ −*ϕ* ^0.33^ predicted by the neo-Hookean model or *Π*_ela_ ∝ −*ϕ* ^0.58^ when accounting for the excluded volume in a good solvent, the nearly linear relation between *Π*_ela_ and *ϕ* agrees with the prediction based on transient entanglements.^17^ ,^18^ Specifically, Obukhov et al. predict that transient entanglements should lead to *Π*_ela_ ∝ −*ϕ* ^0.9^ in a θ-solvent and *Π*_ela_ ∝ −*ϕ* ^1.1^ in a good solvent.^17^ Yamamoto et al. predict that transient entanglements should lead to *Π*_ela_ ∝ −*ϕ*.^18^ These papers assumed neo-Hookean elasticity (eq 4) with a modified effective stretch *λ̂*_i_ = (*R*_0_/*R*)*λ*_i_.^63^ Here, *λ*_i_ is the principal stretches determined from the macroscopic deformation. *R*_0_/*R* is the ratio of the average end-to-end distance of a stress-free chain in the synthesis-state polymer volume fraction *ϕ*_0_ and in the current polymer volume fraction *ϕ*. With this elastic free energy, the swelling-dependent shear modulus *G* reported in these papers has the same scaling as *Π*_ela_ defined by eq 8. Although the prediction based on transient entanglements agrees with the measured *Π*_ela_, these papers claim that all entanglements formed at the synthesis state are permanent; transient entanglements only appear during deswelling.^17,18^ In contrast, our data do not show a noticeable difference between samples deswollen from *ϕ*_0_ = 0.07 and samples swollen from *ϕ*_0_ = 0.41, suggesting that both transient and permanent entanglements form during synthesis. This is reasonable because whether an entanglement is permanently trapped depends on the long-range topology of the chains relative to the cross-linker (Figure 1). Since the cross-linking reaction happens locally, it cannot distinguish between the topology of permanent vs transient entanglements.

In summary, this work uses the constrained swelling test to measure the *Π*_ela_ of polyacrylamide hydrogel synthesized by radical polymerization. The ratios of cross-links and entanglements are systematically varied by controlling the ratio between the cross-linkers and monomers *C* and the polymer volume fraction at synthesis *ϕ*_0_. The measurements show that different ratios of entanglements and cross-links lead to identical *Π*_ela_, suggesting no entanglement sliding during isotropic swelling. This is contrary to existing theories, which assume that entanglements slide under both isotropic and anisotropic deformation. Moreover, the measured *Π*_ela_ of all samples over all ranges shows a nearly linear relation with polymer volume fraction *ϕ*, agreeing with the scaling of transient entanglements. This is contrary to the existing belief that all entanglements are permanently trapped during synthesis, and transient entanglements only form in deswollen polymer networks. Note that this study covers only the intermediate polymer volume fraction. Different behaviors that follow existing theories may appear at the *ϕ* → 0 and *ϕ* → 1 limits.

## Data Availability

The raw data supporting the findings of this study are publicly available at https://doi.org/10.18117/6d98-sx95. The deposited files include primary experimental and/or computational datasets used for analysis. Further information may be obtained from the corresponding author upon reasonable request.
